# Advances in Nutritional Management of Pediatric Inflammatory Bowel Disease

**DOI:** 10.3390/nu13020324

**Published:** 2021-01-23

**Authors:** Wael El-Matary

**Affiliations:** Section of Pediatric Gastroenterology, Winnipeg Children’s Hospital, Max Rady College of Medicine, Rady Faculty of Health Sciences, Children’s Hospital Research Institute, Winnipeg, MB R3A1S1, Canada; welmatary@hsc.mb.ca

Crohn’s disease (CD) and ulcerative colitis (UC) are chronic lifelong non-curable inflammatory bowel diseases (IBD) of uncertain etiology with immune dysfunction likely related to the interaction between the environment and the intestinal microbiome in genetically susceptible individuals. IBD are relatively common and in Canada, they affect 1:140 Canadians [1]. About 8–25% of IBD start before the age of 18 years and the incidence of pediatric IBD is on the rise [2,3]. Complications of IBD include growth restriction and malnutrition through several mechanisms such as anorexia with suboptimal oral intake, nutrient malabsorption, increased intestinal losses, systemic inflammation, and hypermetabolic state [4]. Consequently, nutritional support is an essential therapeutic goal in the care for patients with IBD especially in the pediatric age group. Over the last several decades, there have been very promising animal and human studies shading more light on the nutritional role in correcting IBD-associated intestinal dysbiosis and restoring normal intestinal microbiome. Thus the role of diet, a possible fundamental environmental factor in the pathogenesis of IBD, as a primary therapy for IBD especially in CD has been the focus of many research articles [5]. In this special issue “Advances in Nutritional Management of Pediatric Inflammatory Bowel Disease”, several excellent research and review articles are investigating and highlighting the vital role of diet and nutritional factors in the pathogenesis and treatment of IBD. The issue also contains an interesting case-based discussion highlighting the role of Crohn’s disease exclusion diet (CDED) in treating children with CD.

**Research articles**

Exclusive enteral nutrition (EEN) has been proved effective in induction of clinical remission and mucosal healing in children with active CD [5]. In addition, it may even have a role in maintaining remission if given as supplementary feeds [6]. In a multicenter retrospective analysis [the Prescription of Enteral Nutrition in Paediatric Crohn’s disease (PRESENT) study], Moriczi et al. investigated posible predictors of response of children with active CD to EEN. A total of 83% achieved clinical remission after 6–8 weeks of EEN with 10% of the total cohort taking the formula via nasogastric tube. Patients with mild to moderate CD with weighed pediatric CD activity index ≤ 57.5, fecal calprotectin < 500 μg/g, and C-reactive protein (CRP) >15 mg/L tended to respond better to EEN. Numerically more patients with ileal disease responded to EEN (*p* = 0.064) but ileal disease was a predictive factor of response to EEN in a multivariable model (*p* = 0.039) [7]. The retrospective design and lack of uniform approach among included centers may have limited the study conclusions.

Hart et al. investigated the effect of EEN versus corticosteroids on the intestinal microbiome of small cohort of children with active IBD. They found no difference in the fecal microbiota between the 2 modalities of treatment when patients achieve clinical remission. i.e., while there is a significant difference in the fecal microbiota between patients with active disease versus those who achieved remission, the choice of treatment did not lead to a different microbiome in those who achieved clinical remission [8]. The study was limited by the small sample size.

In a randomized controlled study, Suskind et al. examined the efficacy of 3 different versions of specific carbohydrate diet (SCD) in inducing remission in 18 children (7–18 y) with mild to moderately active CD with 10 patients on medications including immunomodulators and biologics. Only 10 of 18 patients completed the 12-week study and achieved clinical remission [9]. The small sample size, effect of medications, participants own preference of food elements and inability to accurately assess adherence to SCD were the main limitations of the study.

In a cohort study, Rempel et al. assessed the frequency of anemia and micronutrient deficiencies in children with IBD at diagnosis and after one year of treatment. The prevalence of deficiencies in our cohort at diagnosis and one-year follow-up, respectively were iron (56% and 27%), ferritin (39% and 27%), zinc (10% and 6%), vitamin D (22% and 13%), vitamin A (25% and 25%), vitamin E (5% and 4%), and selenium (10 and 7%). Anemia was present in 57% and 25% at diagnosis and follow up respectively. Age of diagnosis (15–18 y) and low serum albumin levels (<33 g/L) were predictors of moderate to severe anemia at diagnosis in children with CD [10].

The study was limited by the lack of a control group.

Strisciuglio et al. explored the role of diet in maintaining clinical remission in children with IBD. They evaluated dietary intake in 125 children with IBD (72 with UC and 53 with CD) and similar number of healthy controls without IBD using a 3-day food diary and the Mediterranean Diet Quality Index for Children and Adolescents (KIDMED). They found significant differences in carbohydrate and protein intake between children with IBD and healthy controls. They also noticed some differences between those with CD compared to those with UC in protein, vitamin D and iron intake. In children with IBD, adherence to the Mediterranean diet was associated with low levels of fecal calprotectin [11]. The investigators recommended routine screening of patients with IBD for nutritional inadequacy.

**Review articles**

In an excellent review, Konstantinos et al. summarized the available evidence on dietary interventions promoting maintenance of remission in IBD. While there are some promising results with some diets such as the CDED and Mediterranean diet, there has not been enough evidence to recommend a specific diet for patients with IBD to maintain remission yet. Even with EEN that was proved to be effective for induction of remission in active CD, its efficacy to maintain remission when it is used as supplementary (MEN) to normal diet has not been established yet although assessing compliance with nutritional formula in these studies is a challenge. With introduction of normal diet, inactive IBD usually relapse. The review provides a summary of literature on how to introduce regular food elements with a questionable clinical efficacy of some exclusion diets. The authors recommended more well-planned adequately powered controlled studies with monitoring colonic mucosal inflammation before specific food re-introduction protocols are routinely implemented in clinical care. Out of all the different food-based strategies explored in that review, CDED + MEN, the semi-vegetarian diet and the carrageenan-free diet demonstrated signals of efficacy in maintaining clinical remission. However, these signals were mostly associated with maintenance of clinical disease activity and symptomatic improvement, rather than control of gut-specific inflammation. On the other hand, there is no strong evidence that certain diets are associated with high risk of relapse. The authors concluded that while there are reasons to believe that there are specific dietary triggers of gut inflammation in IBD, those triggers will need to be identified through high-quality dietary studies [12]. 

Mentella et al. reviewed the literature evaluating different diets such as SCD, low fermentable oligosaccharides, disaccharides, monosaccharides and polyols (FODMAP) diet, gluten free diet, anti-inflammatory diet and Mediterranean diet are investigated with regard to their impact on microbiota and on the evolution of the disease. The effects of different diets on IBD seemed to be mediated through their effect on intestinal microbiome. However, the authors concluded that currently there is no clear indication toward a specific diet for managing IBD but they ambitiously recommended the assessment of dysbiosis prior to the recommendation of a specific diet to become a standard clinical approach in order to achieve a personalized therapy [13].

In their review, Ghiboub et al. covered an interesting topic and described recent advances of tryptophan (Trp) metabolism and the aryl hydrocarbon receptor (AhR) signaling in human health and disease including colitis, with a focus on nutrition as a potential therapy to modulate Trp metabolites acting on AhR. The authors highlighted some positive results of AhR activation in experimental colitis and discussed the evidence of the role of curcumin in the management of IBD. Promising potentials of nutritional modification of AhR signaling may exist [14].

Another topic that was nicely covered by Healey et al. was the role of microbiome-targeted therapeutics such as prebiotics and fibers in IBD through their positive effect on the intestinal microbiome. The review discussed the mechanisms by which prebiotics and fibers impact the microbiome. The authors highlighted an important knowledge gap concerning the absence of studies investigating the role of prebiotics and fibers as potential therapeutic agents for pediatric IBD [15]. On the other hand, there are several papers that showed some efficacy of fibers and prebiotics in improving IBD-associated symptoms [16,17]. Moreover, a recent meta-analysis that investigated the association between fiber intake and the risk of developing IBD which included studies with children and adolescents showed a significant inverse relationship between high fiber intake and the risk of developing CD [18]. However, this risk was marginally significant for UC.

**A cased-based discussion**

Finally, the issue included a cased-based discussion concerning clinical and practical aspects of using CDED. Thus far and based on the current evidence, CDED is arguably the most promising diet in treating mild to moderate CD after EEN but with a better tolerance and palatability than EEN. The illustrative cases discussed provided some guidance to practitioners on the context and clinical scenarios of using CDED alone, with EEN, or with partial enteral nutrition (PEN). In order to improve adherence, a multidisciplinary team approach is most effective. Clinical care pathways can help to optimize communication with patients and families as well as multidisciplinary decision-making [19]. Online resources of nutrients and recipes for each phase have been developed and can help in facilitating communication between caregivers and patients (mymodulife.com, modulifexpert.com).
